# Supplementary material for: Exploring the impact of primer length on efficient gene detection via high-throughput sequencing
Source: Nat Commun. 2024 Jul 12;15:5858. doi: 10.1038/s41467-024-49685-0 (PMC11245535; doi:10.1038/s41467-024-49685-0)
Supplement: Supplementary file 1 — Supplementary Information [file 41467_2024_49685_MOESM1_ESM.pdf]

Exploring the Impact of Primer Length on Efficient Gene Detection via High-Throughput Sequencing

Author list

Julia Micheel, Aram Safrastyan, Franziska Aron, Damian Wollny

Supporting Information

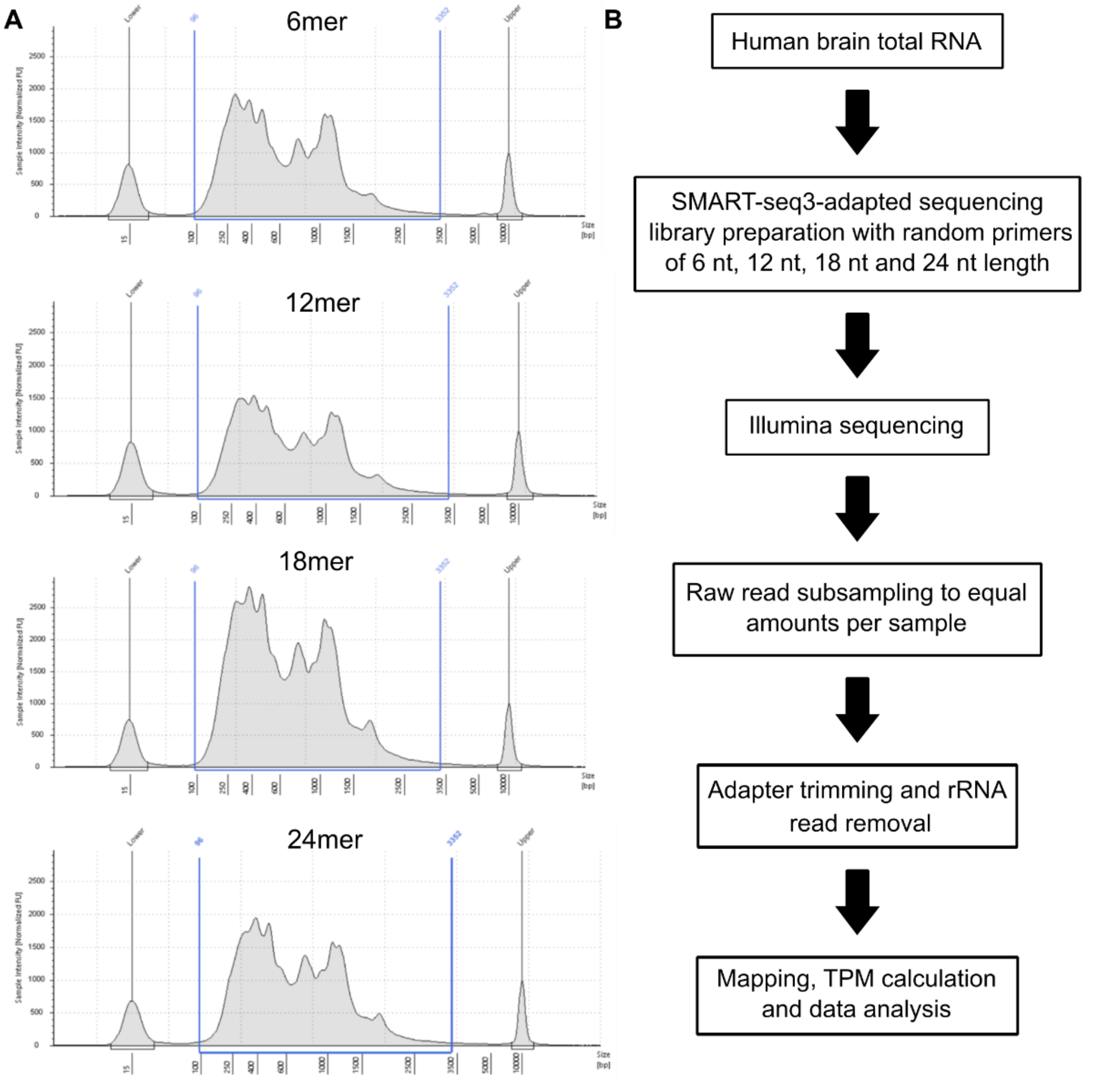

Supplementary Figure 1: **Workflow of RNA sequencing data generation. a** Fragment length distribution of the pre-amplified cDNAs generated with random primers of different lengths, one exemplary TapeStation plot per primer. **b** Schematic of the workflow from sequencing library generation to data analysis.

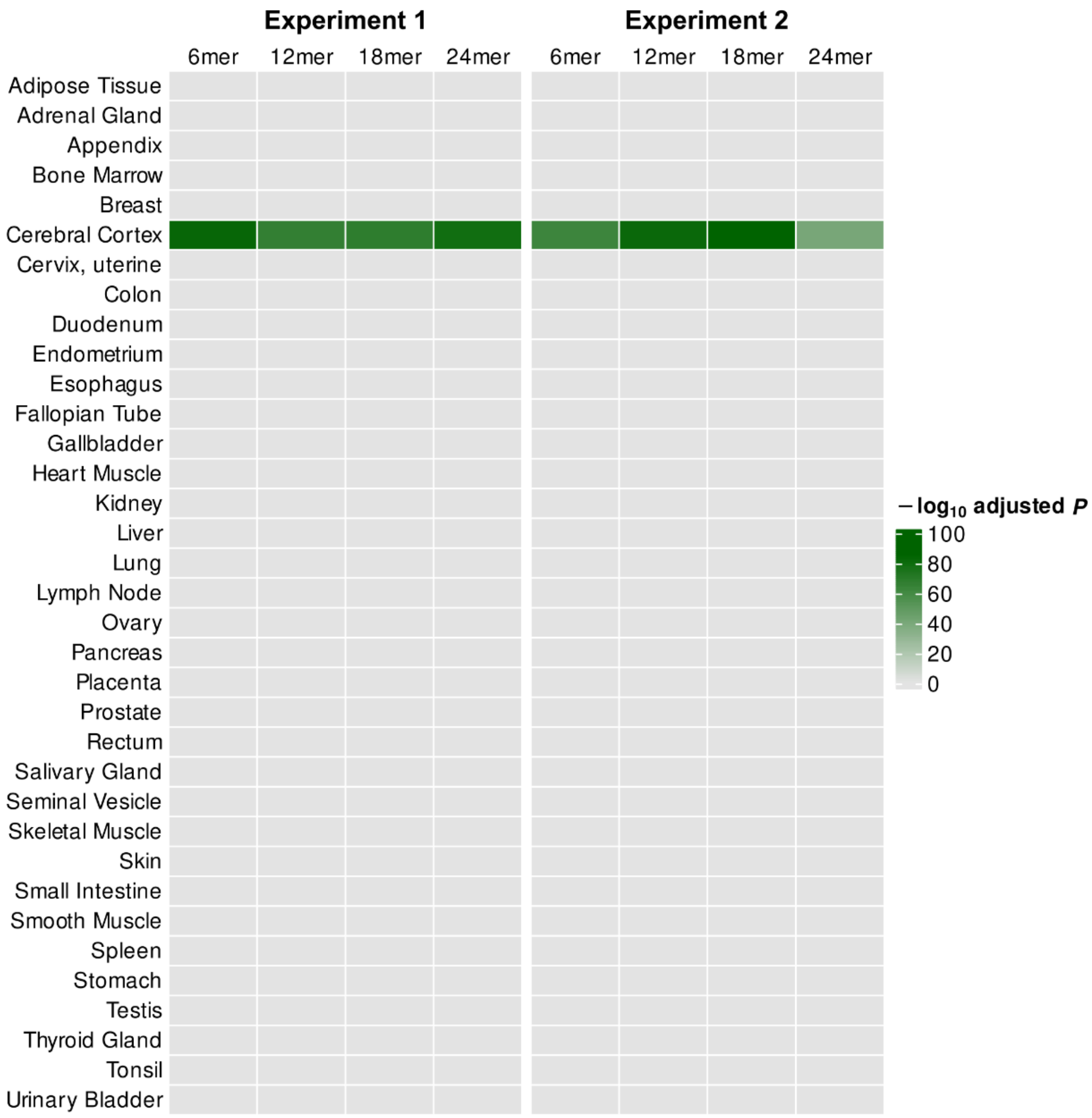

Supplementary Figure 2: **Tissue enrichment analysis.** Tissue enrichment analysis was carried out for genes detected by each primer (experiment 1: 5 million reads, experiment 2: 30 million reads; both n = 3 technical replicates). Genes of the categories "tissue-enriched", "group-enriched" and "tissue-enhanced" according to Human Protein Atlas were considered as tissue-specific genes. The resulting *p*-values were adjusted for multiple comparisons using the Benjamini-Hochberg method and $-\log_{10}$ transformed. Source data is provided as a Source Data file.

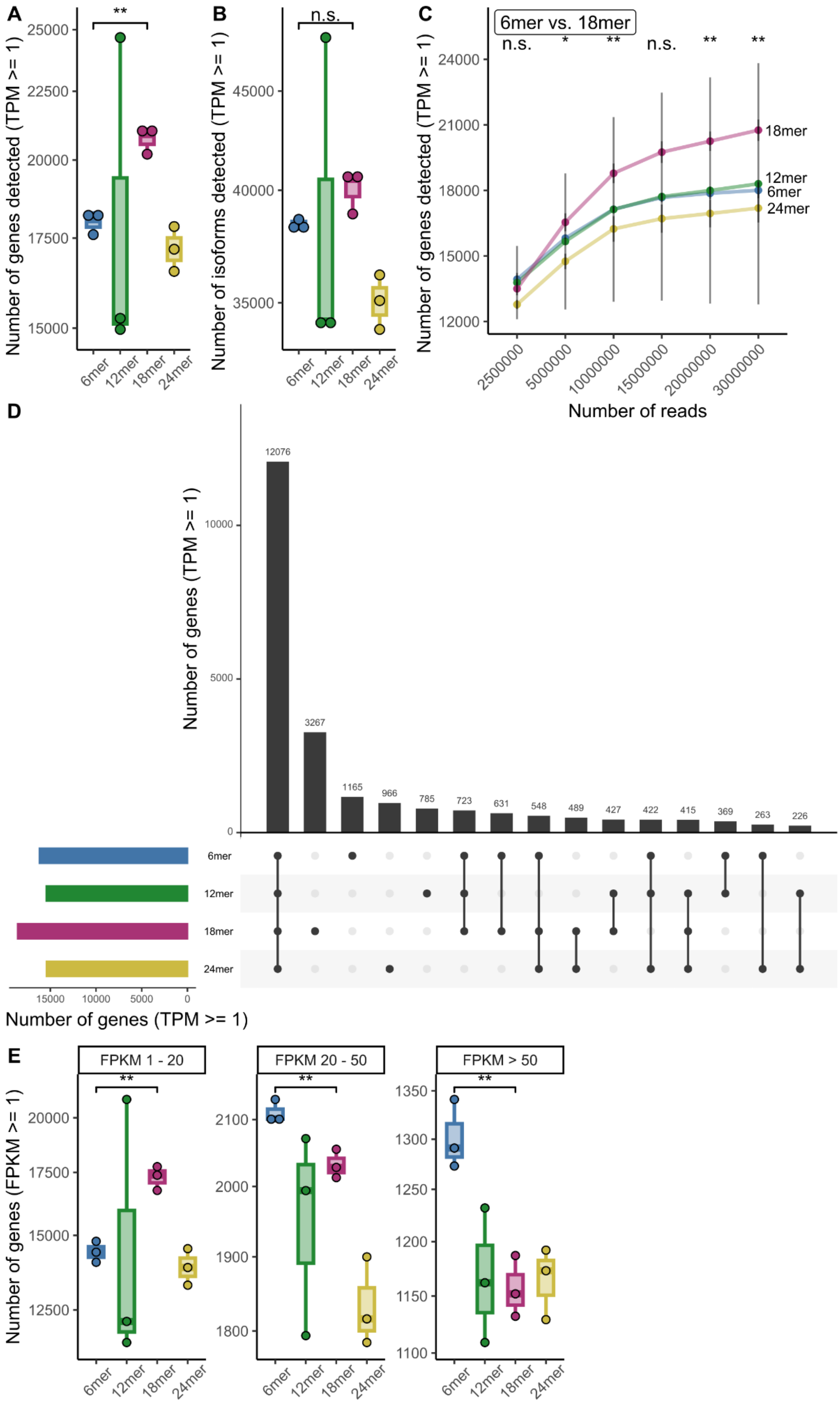

A
B
C
D
E
Number of genes detected (TPM >= 1)
Number of isoforms detected (TPM >= 1)
6mer vs. 18mer
n.s.
18mer
12mer
6mer
24mer
Number of reads
Number of genes (TPM >= 1)
12076
3267
1165
966
785
723
631
548
489
427
422
415
369
263
226
FPKM 1 - 20
FPKM 20 - 50
FPKM > 50
Number of genes (FPKM >= 1)

Supplementary Figure 3: **Quantification of the detected genes by primers of varying length after high depth RNA sequencing. a** The numbers of genes detected (TPM >= 1) and **b** the number of isoforms detected (TPM >= 1) from 30 million raw reads per sample are depicted as box plots (n = 3 technical replicates). **c** Quantification of gene detection after random subsampling (2.5 - 30 million raw reads) is shown. n = 3 technical replicates; error bars = mean ± standard deviation. **d** Intersections of the genes detected using the random primers of different lengths. Here, all genes were considered per primer that were found in at least two of the three replicates after computational subsampling to 30 million reads. The total numbers of genes detected per random primer fulfilling this criteria are depicted as an additional bar chart on the left. **e** The numbers of detected genes (FPKM >= 1) per FPKM bin are depicted as box plots (30 million reads, n = 3 technical replicates). Box plots display the interquartile range (25th - 75th percentiles); center line = median; whiskers extend to the largest (maxima) and smallest (minima) values within 1.5 x interquartile range. *$p < 0.05$, **$p < 0.01$, n.s. = not significant. Source data is provided as a Source Data file. Statistical tests (unpaired two-sided Student's *t*-test and Mann-Whitney *U*-test) performed in a, b, c and e with corresponding *p*-values are reported in Supplementary Data 1.

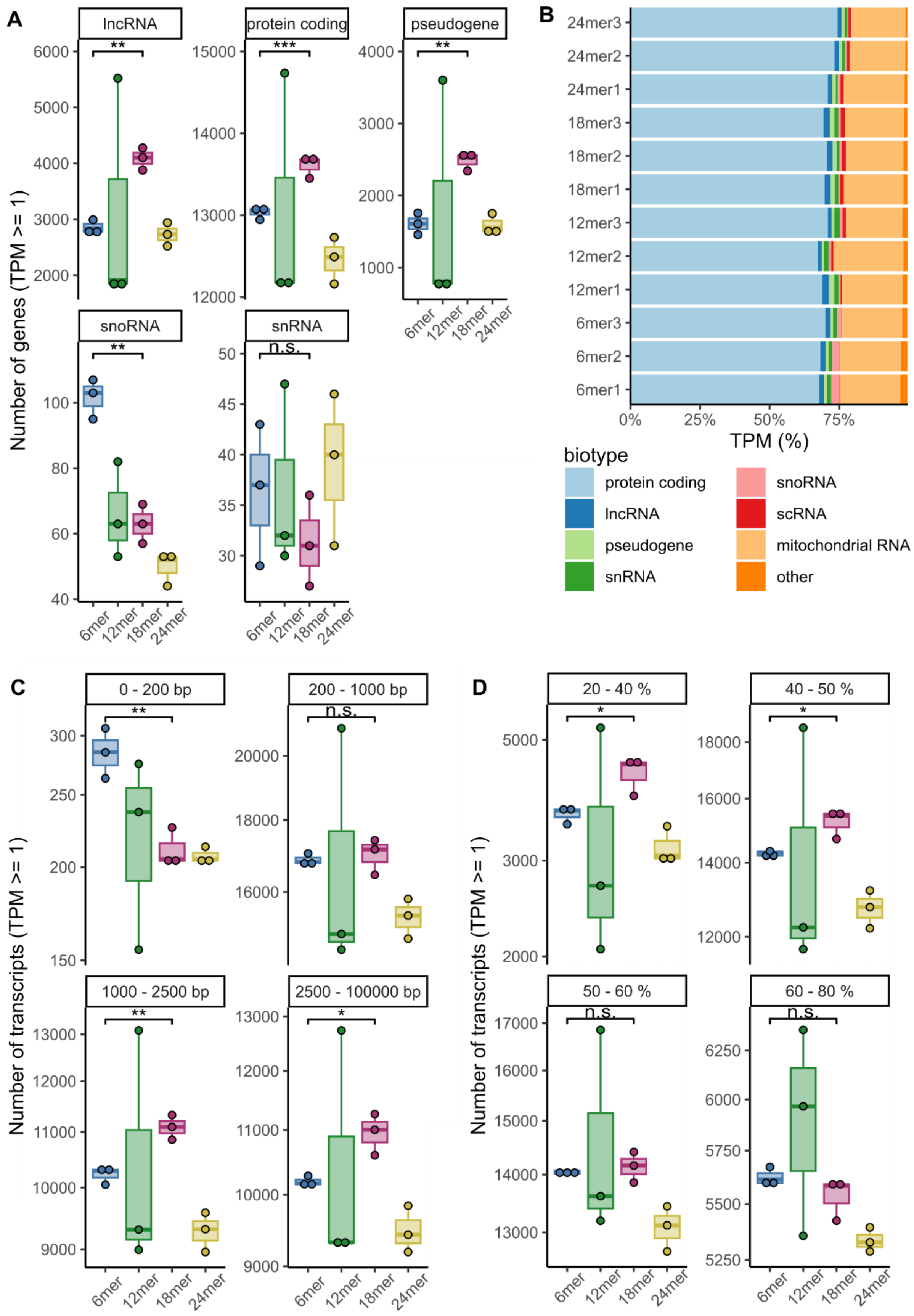

Supplementary Figure 4: **Characterization of detected genes by primers of varying lengths after high depth RNA sequencing. a** The numbers of detected genes (TPM >= 1) per biotype (30 million reads, n = 3 technical replicates). **b** Transcript proportions of the

different biotypes detected per sample (subsampled to 30 million reads). **c** The numbers of detected transcripts (TPM >= 1) per transcript length are depicted as box plots (30 million reads, n = 3 technical replicates). In the box plots, the box represents the interquartile range between the 25th and 75th percentiles with the center line denoting the median; the whiskers extend to the largest (maxima) and smallest (minima) values within 1.5 times the interquartile range. **d** The numbers of detected transcripts (TPM >= 1) per transcript GC content are depicted as box plots (5 million reads, n = 3 technical replicates). Box plots display the interquartile range (25th - 75th percentiles); center line = median; whiskers extend to the largest (maxima) and smallest (minima) values within 1.5 x interquartile range. *$p < 0.05$, **$p < 0.01$, ***$p < 0.001$, n.s. = not significant. Source data is provided as a Source Data file. Statistical tests (unpaired two-sided Student's *t*-test and Mann-Whitney *U*-test) performed in a, c and d with corresponding *p*-values are reported in Supplementary Data 1.

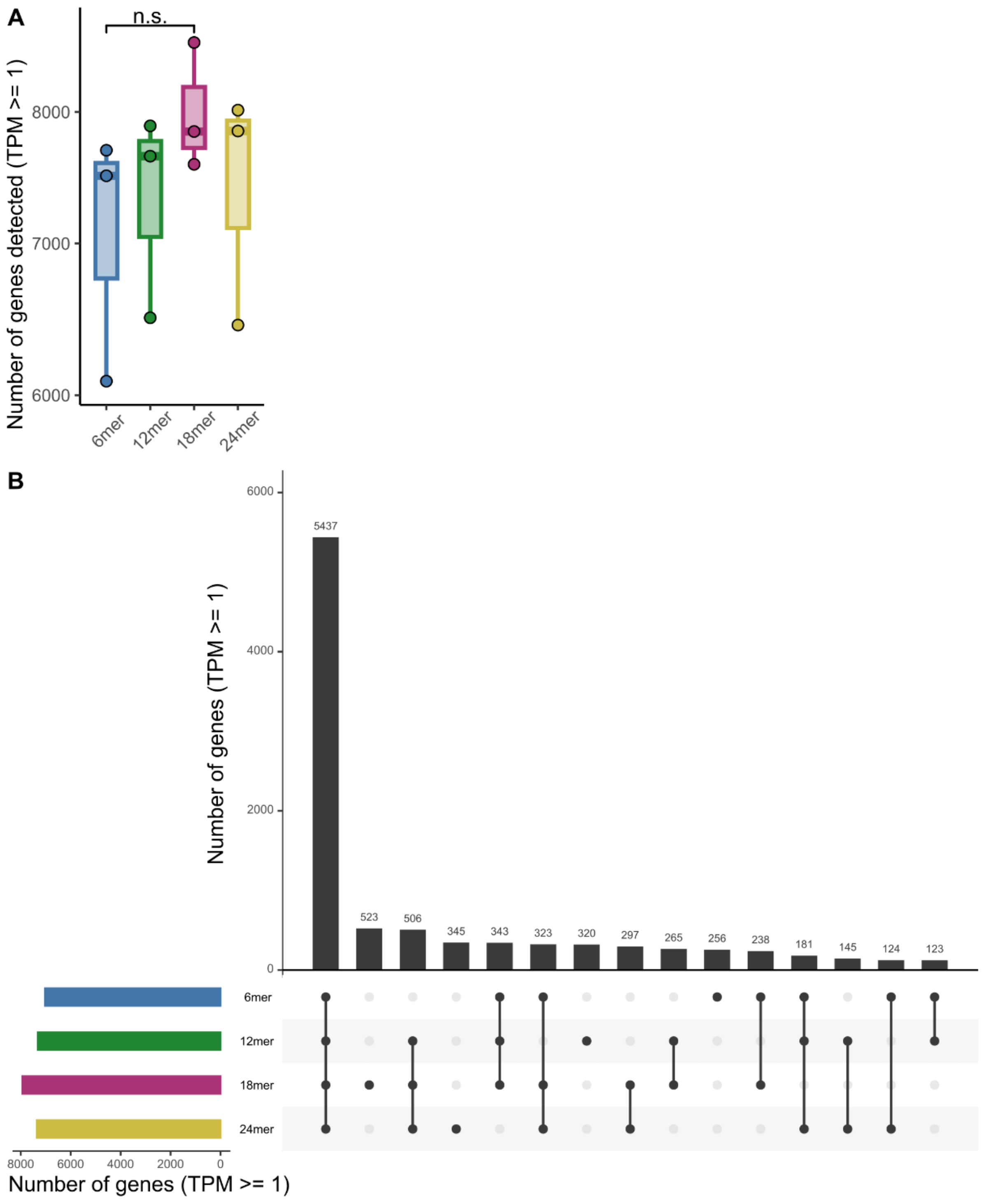

Supplementary Figure 5: **Quantification of the detected genes by primers of varying length from SARS-CoV-2-infected Vero cells. a** The numbers of genes detected (TPM >= 1) from 7 million raw reads per technical triplicate are depicted as box plots (n = 3 technical replicates). In the box plot, the box represents the interquartile range between the 25th and 75th percentiles with the center line denoting the median; the whiskers extend to the largest (maxima) and smallest (minima) values within 1.5 times the interquartile range. Statistical significance was determined using the unpaired two-sided Student's *t*-test; n.s. = not significant. The statistical significance test performed on this data together with corresponding exact *p*-value is reported in Supplementary Data 1. **b** Intersections of the genes detected using

the random primers of different lengths. Here, all genes were considered per primer that were found in at least two of the three replicates (7 million reads). The total numbers of genes detected per random primer fulfilling this criteria are depicted as an additional bar chart on the left. Source data is provided as a Source Data file.

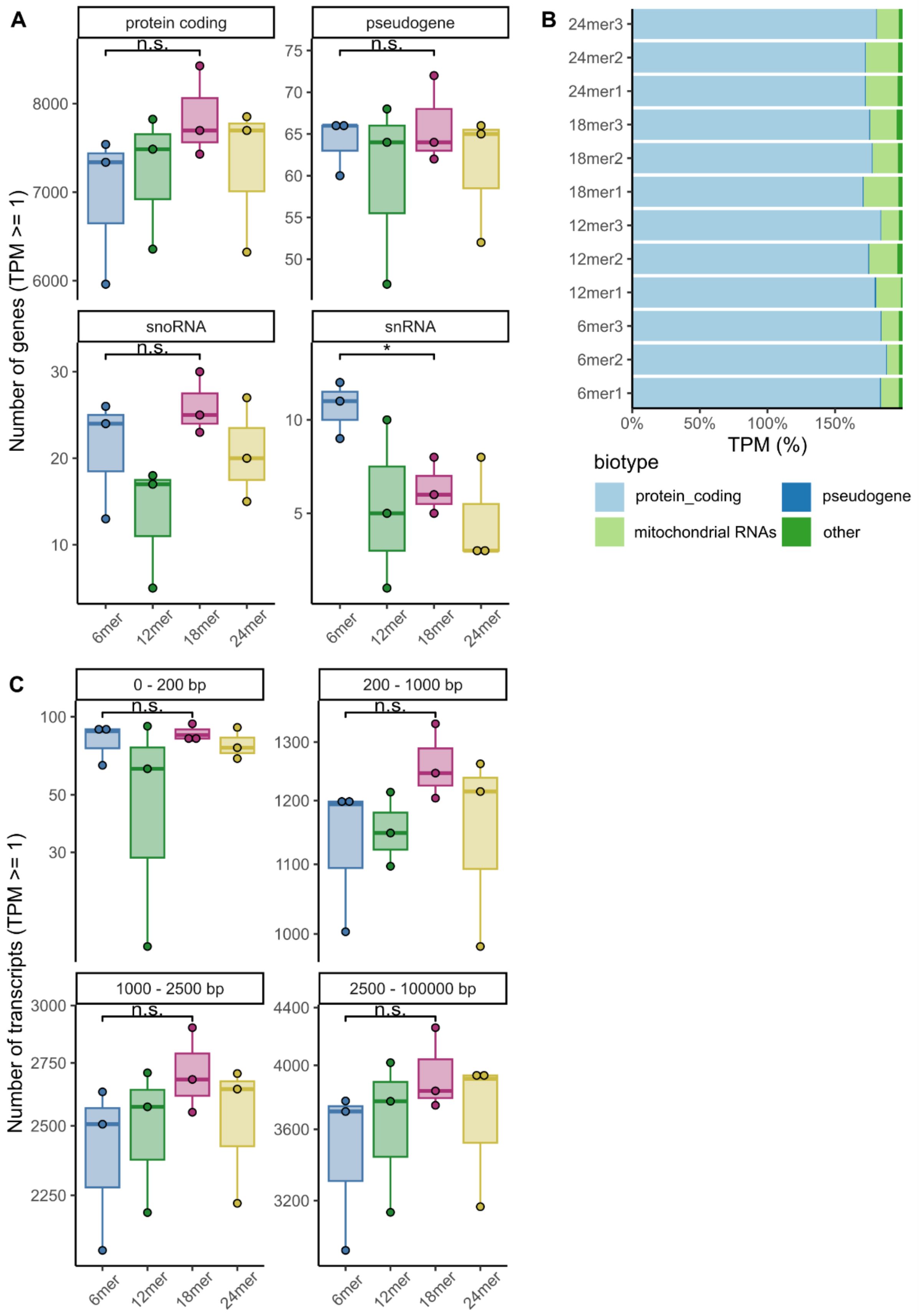

Supplementary Figure 6: **Characterization of detected genes by primers of varying length from SARS-CoV-2-infected Vero cells. a** The numbers of detected genes (TPM >= 1) per

biotype (7 million reads, n = 3 technical replicates). **b** Transcript proportions of the different biotypes detected per sample (subsampled to 7 million reads). **c** The numbers of detected transcripts (TPM >= 1) per transcript length are depicted as box plots (7 million reads, n = 3 technical replicates). Box plots display the interquartile range (25th - 75th percentiles); center line = median; whiskers extend to the largest (maxima) and smallest (minima) values within 1.5 x interquartile range. *$p < 0.05$, n.s. = not significant. Source data is provided as a Source Data file. Statistical tests (unpaired two-sided Student's *t*-test and Mann-Whitney *U*-test) performed in a and c with corresponding *p*-values are reported in Supplementary Data 1.

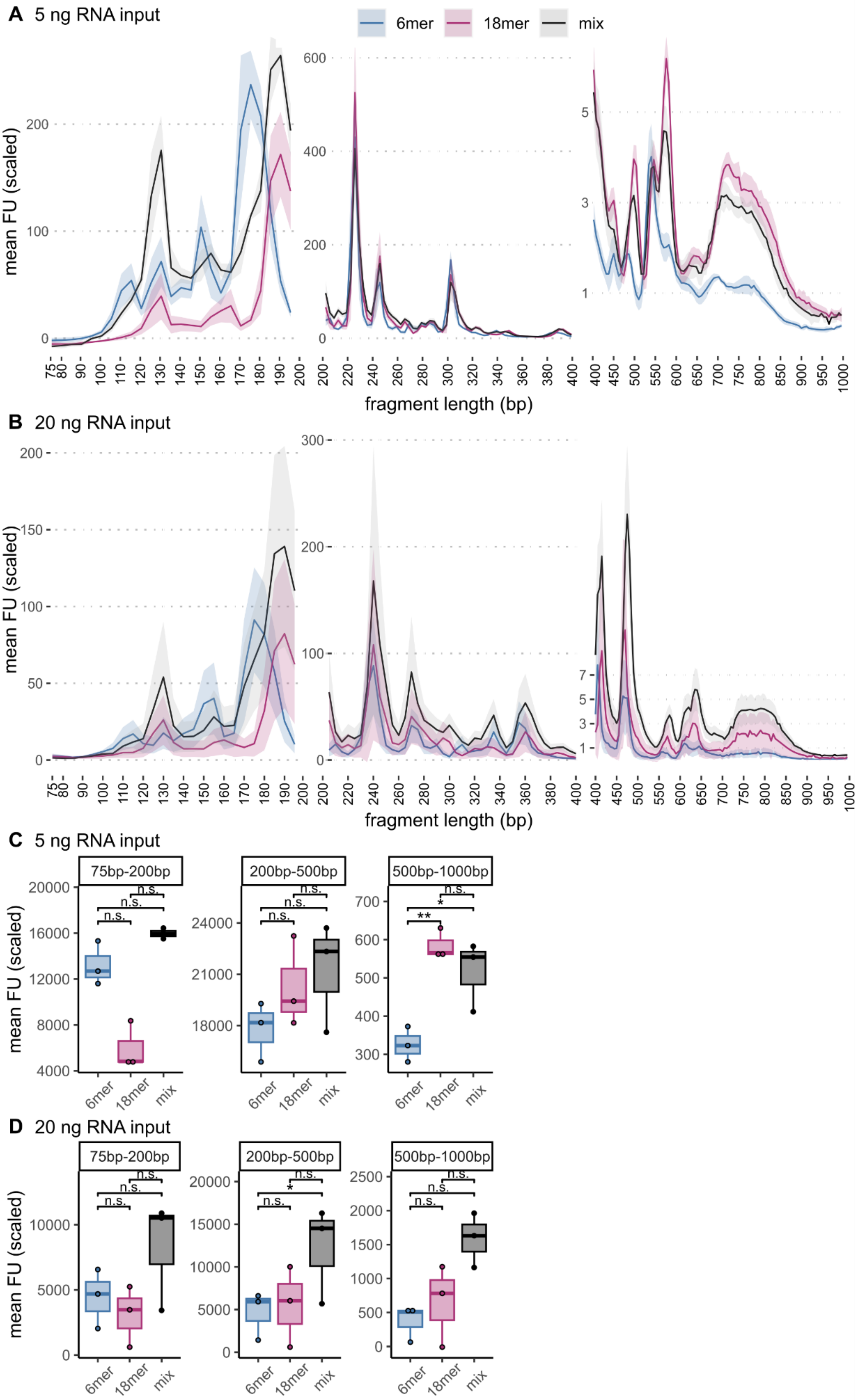
A 5 ng RNA input
6mer
18mer
mix
mean FU (scaled)
fragment length (bp)
B 20 ng RNA input
mean FU (scaled)
fragment length (bp)
C 5 ng RNA input
75bp-200bp
200bp-500bp
500bp-1000bp
mean FU (scaled)
n.s.
*
**
6mer
18mer
mix
D 20 ng RNA input
75bp-200bp
200bp-500bp
500bp-1000bp
mean FU (scaled)
n.s.
*
6mer
18mer
mix

Supplementary Figure 7: **Characterization of cDNA synthesized from artificial RNA input with random primers of varying length.** Input RNA of 50, 80, 150, 300, 500 and 1000 nt in length was used as input material for reverse transcription. Random primers of 6 nt, 18 nt and a mixture of both (mix) were used. The pre-amplified cDNA fragment profiles were analyzed on the Bioanalyzer using a High Sensitivity DNA kit. Mean fluorescence lines with standard deviation ribbons from three technical replicates are shown for the cDNAs generated from **a** 5 ng input RNA and **b** 20 ng input RNA. Quantification of the scaled fluorescence per fragment length bin are shown as box plots for cDNAs generated from **c** 5ng input RNA and **d** 20 ng input RNA (n = 3 technical replicates). In the box plots, the box represents the interquartile range between the 25th and 75th percentiles with the center line denoting the median; the whiskers extend to the largest (maxima) and smallest (minima) values within 1.5 times the interquartile range. Statistical significance was determined using the unpaired two-sided Student's *t*-test and the two-sided Mann-Whitney *U*-test with adjustment for multiple comparisons using the Benjamini-Hochberg method; *$p < 0.05$, **$p < 0.01$, n.s. = not significant. Source data are provided as a Source Data file. Statistical significance tests performed on the data shown in c and d together with corresponding exact *p*-values are reported in Supplementary Table 4.

Supplementary Table 1: **Expected expression in the brain of all detected protein-coding genes.** Protein-coding genes were classified as expressed in brain or other tissue according to the Human Protein Atlas and are displayed as total numbers and proportions.

**Experiment**	**Sample**	**Expressed in brain**	**Not expressed in brain**	**Expressed in brain (%)**	**Not expressed in brain (%)**
Experiment 1 (5 million reads)	6mer1	7353	145	98.07	1.93
	6mer2	7918	158	98.04	1.96
	6mer3	7605	141	98.18	1.82
	12mer1	8734	186	97.91	2.09
	12mer2	7768	172	97.83	2.17
	12mer3	8009	168	97.95	2.05
	18mer1	9753	236	97.64	2.36
	18mer2	8797	173	98.07	1.93
	18mer3	9763	262	97.39	2.61
	24mer1	7150	133	98.17	1.83
	24mer2	8488	156	98.20	1.80
	24mer3	8225	143	98.29	1.71
Experiment 2 (30 million reads)	6mer1	12526	543	95.85	4.15
	6mer2	12573	501	96.17	3.83
	6mer3	12498	447	96.55	3.45
	12mer1	13555	1180	92.00	8.01
	12mer2	11927	252	97.93	2.07
	12mer3	11893	277	97.73	2.28
	18mer1	12796	867	93.65	6.35
	18mer2	12644	807	94.00	6.00
	18mer3	12813	892	93.50	6.51
	24mer1	12164	565	95.56	4.44
	24mer2	12018	473	96.21	3.79
	24mer3	11743	427	96.49	3.51

Supplementary Table 2: **Expected expression in the brain of the uniquely detected protein-coding genes.** Protein-coding genes that were uniquely detected per primer and experiment were classified as expressed in brain or other tissue according to the Human Protein Atlas and are displayed as total numbers and proportions.

Experiment	Sample	Expressed in brain	Not expressed in brain	Expressed in brain (%)	Not expressed in brain (%)
Experiment 1 (5 million reads)	6mer	299	16	94.92	5.08
	12mer	405	26	93.97	6.03
	18mer	979	47	95.42	4.58
	24mer	402	13	96.87	3.13
Experiment 2 (30 million reads)	6mer	287	72	79.94	20.06
	12mer	197	50	79.76	20.24
	18mer	531	347	60.48	39.52
	24mer	172	60	74.14	25.86

Supplementary Table 3: **Functional annotation of the uniquely detected genes per primer.** Genes that were uniquely detected with one primer length in at least two out of three technical replicates (Supplementary Data 3 - 6 and 8 - 11) were analyzed for functional enrichment using the DAVID Functional Annotation tool.[44,45] All genes that were mapped by DAVID were analyzed for functional annotation (Count threshold: 2, EASE threshold: 0.1).[44,45] Significant functional enrichment was defined as Benjamini-Hochberg adjusted *p*-value <= 0.05. The numbers of unique genes per category as well as the number of pathways with significant enrichment are shown in the table.

	**Primer**	**Uniquely detected genes**	**Mapped by DAVID**	**Passed count and EASE threshold**	**Genes with significant functional enrichment (Benjamini-Hochberg <= 0.05)**	**Number of pathways with significant enrichment**
Experiment 1 (5 million reads)	6mer	505	393	228	49 (9.7%)	6
	12mer	643	512	398	0 (0.0%)	0
	18mer	1423	1181	1019	776 (54.5%)	4
	24mer	627	482	398	174 (20.3%)	23
Experiment 2 (30 million reads)	6mer	1165	681	281	30 (2.6%)	1
	12mer	785	381	142	0 (0.0%)	0
	18mer	3267	1808	723	589 (18.0%)	60
	24mer	966	513	170	93 (9.6%)	4

Supplementary Table 4: **Statistical analyses of the electrophoresis experiment.** Summary of the statistical tests performed to analyze whether the differences between 6mer, 18mer and mixed samples are significant. First, normal distribution and variance equality of the respective data were tested and based on that the statistical significance test was chosen: a two-sided Student's *t*-test in case of normal distribution and variance equality or a Mann-Whitney *U*-test. The respective chosen test and its result are shown in this table. More details about the normal distribution and variance equality test are shown in Supplementary Data 1. Per RNA input amount (5 ng, 20 ng), 3 technical replicates starting from input RNA were produced and analyzed.

**Data set**	**5 ng artificial RNA, n = 3**			**20 ng artificial RNA, n = 3**		
**comparison**	**6mer vs 18mer**	**6mer vs mix**	**18mer vs mix**	**6mer vs 18mer**	**6mer vs mix**	**18mer vs mix**
**75 - 200 bp**	Figure S7c			Figure S7d		
Stat. test	Mann-Whitney *U* test	*t*-test	Mann–Whitney *U* test	*t*-test	*t*-test	*t*-test
p-value	0.1000	0.0719	0.1000	0.8180	0.1900	0.1900
**200 - 500 bp**	Figure S7c			Figure S7d		
Stat. test	*t*-test	*t*-test	*t*-test	*t*-test	*t*-test	*t*-test
p-value	0.4250	0.4250	0.6730	0.4770	0.0474 (*)	0.0617
**500 - 1000 bp**	Figure S7c			Figure S7d		
Stat. test	*t*-test	*t*-test	*t*-test	*t*-test	*t*-test	*t*-test
p-value	0.00737 (**)	0.0156 (*)	0.2330	0.6170	0.2640	0.2550

Supplementary Table 5: **Relationship between reverse transcription efficiency and gene detection.** Concentrations of the cDNAs generated after reverse transcription and pre-amplification in the Vero cell experiment are shown as well as the quantification of total genes detected per technical replicate.

	6mer		12mer		18mer		24mer	
	**ng/µl**	**total genes detected**	**ng/µl**	**total genes detected**	**ng/µl**	**total genes detected**	**ng/µl**	**total genes detected**
replicate 1	7.92	7695	11.9	7888	13.2	8585	8.92	8015
replicate 2	2.90	6086	6.92	7649	5.86	7586	7.44	7847
replicate 3	7.22	7498	3.44	6491	7.72	7843	2.84	6443

Supplementary Table 6: **Text mining analysis.** Text mining was performed via the PMC Open Access Subset using the search terms shown in the table yielding the respective numbers of publications. Data was retrieved on 24.02.2022.

Search terms	# publications
((("reverse transcription") AND "random tetramer") OR "random 4-mer") OR "random 4mer"	4
((("reverse transcription") AND "random pentamer") OR "random 5-mer") OR "random 5mer"	6
((("reverse transcription") AND "random hexamer") OR "random 6-mer") OR "random 6mer"	11519
((("reverse transcription") AND "random heptamer") OR "random 7-mer") OR "random 7mer"	9
((("reverse transcription") AND "random octamer") OR "random 8-mer") OR "random 8mer"	26
((("reverse transcription") AND "random nonamer") OR "random 9-mer") OR "random 9mer"	53
((("reverse transcription") AND "random decamer") OR "random 10-mer") OR "random 10mer"	102
((("reverse transcription") AND "random undecamer") OR "random 11-mer") OR "random 11mer"	0
((("reverse transcription") AND "random dodecamer") OR "random 12-mer") OR "random 12mer"	25
((("reverse transcription") AND "random tridecamer") OR "random 13-mer") OR "random 13mer"	3
((("reverse transcription") AND "random tetradecamer") OR "random 14-mer") OR "random 14mer"	4
((("reverse transcription") AND "random pentadecamer") OR "random 15-mer") OR "random 15mer"	17
((("reverse transcription") AND "random hexadecamer") OR "random 16-mer") OR "random 16mer"	3
((("reverse transcription") AND "random heptadecamer") OR "random 17-mer") OR "random 17mer"	0
((("reverse transcription") AND "random octadecamer") OR "random 18-mer") OR "random 18mer"	4
((("reverse transcription") AND "random nonadecamer") OR "random 19-mer") OR "random 19mer"	3
((("reverse transcription") AND "random icosamer") OR "random 20-mer") OR "random 20mer"	18
((("reverse transcription") AND "random henicosamer") OR "random 21-mer") OR "random 21mer"	4
((("reverse transcription") AND "random docosamer") OR "random 22-mer") OR "random 22mer"	3
((("reverse transcription") AND "random tricosamer") OR "random 23-mer") OR "random 23mer"	1
((("reverse transcription") AND "random tetracosamer") OR "random 24-mer") OR "random 24mer"	1
