# Supplementary material for: Exploring the impact of primer length on efficient gene detection via high-throughput sequencing
Source: Nat Commun. 2024 Jul 12;15:5858. doi: 10.1038/s41467-024-49685-0 (PMC11245535; doi:10.1038/s41467-024-49685-0)
Supplement: Supplementary file 3 — Description of Additional Supplementary Files [file 41467_2024_49685_MOESM3_ESM.pdf]

Description of Additional Supplementary Files

Supplementary Data 1: Statistical analyses of the sequencing experiments. Summary of the statistical tests performed to analyze whether the differences between 6mer and 18mer samples are significant. First, normal distribution and variance equality of the respective data were tested and based on that the statistical significance test was chosen: two-sided Student's *t*-test in case of normal distribution and variance equality or two-sided Mann-Whitney *U*-test. The respective chosen test and its result are shown in this table. More details about the normal distribution and variance equality test are shown in Supplementary Data 2. For each data set, 3 technical replicates starting from input RNA were produced and analyzed.

Supplementary Data 2: Statistical test details summarized for all experiments. Statistical significance of differences between 6mer and 18mer were tested as shown in Supplementary Data 1 as well as between 6mer, 18mer and a mix of both primers as shown in Supplementary Table 4. First, normal distribution and variance equality of the respective data were tested. The results are shown in this data frame. Based on that the statistical significance test was chosen: a two-sided Student's *t*-test in case of normal distribution and variance equality or a Mann-Whitney *U*-test. The respective chosen test, its result and the effect size are shown in this table.

Supplementary Data 3: Uniquely detected genes of the random 6mer. Ensembl gene identifiers, gene names, biotypes as well as functional information of the genes that were uniquely detected with the random 6mer in at least two out of three technical replicates (sequencing experiment 1, subsampled to 5 million reads per replicate, n = 3).

Supplementary Data 4: Uniquely detected genes of the random 12mer. Ensembl gene identifiers, gene names, biotypes as well as functional information of the genes that were uniquely detected with the random 12mer in at least two out of three technical replicates (sequencing experiment 1, subsampled to 5 million reads per replicate, n = 3).

Supplementary Data 5: Uniquely detected genes of the random 18mer. List of Ensembl gene identifiers, gene names, biotypes as well as functional information of the genes that were uniquely detected with the random 18mer in at least two out of three technical replicates (sequencing experiment 1, subsampled to 5 million reads per replicate, n = 3).

Supplementary Data 6: Uniquely detected genes of the random 24mer. List of Ensembl gene identifiers, gene names, biotypes as well as functional information of the genes that were uniquely detected with the random 24mer in at least two out of three technical replicates (sequencing experiment 1, subsampled to 5 million reads per replicate, n = 3).

Supplementary Data 7: Significant pathway enrichment of the uniquely detected genes. Pathway enrichment analysis of the uniquely detected genes per primer was performed using DAVID.[44,45] Significantly enriched (Benjamini Hochberg <= 0.05) pathways per primer are listed.

Supplementary Data 8: Uniquely detected genes of the random 6mer. Ensembl gene identifiers, gene names, biotypes as well as functional information of the genes that were

uniquely detected with the random 6mer in at least two out of three technical replicates (sequencing experiment 2, subsampled to 30 million reads per replicate, n = 3).

Supplementary Data 9: Uniquely detected genes of the random 12mer. Ensembl gene identifiers, gene names, biotypes as well as functional information of the genes that were uniquely detected with the random 12mer in at least two out of three technical replicates (sequencing experiment 2, subsampled to 30 million reads per replicate, n = 3).

Supplementary Data 10: Uniquely detected genes of the random 18mer. Ensembl gene identifiers, gene names, biotypes as well as functional information of the genes that were uniquely detected with the random 18mer in at least two out of three technical replicates (sequencing experiment 2, subsampled to 30 million reads per replicate, n = 3).

Supplementary Data 11: Uniquely detected genes of the random 24mer. Ensembl gene identifiers, gene names, biotypes as well as functional information of the genes that were uniquely detected with the random 24mer in at least two out of three technical replicates (sequencing experiment 2, subsampled to 30 million reads per replicate, n = 3).

Supplementary Data 12: Ordering details of the consumables. Names, companies and article numbers of all consumables described in the Methods section.
