# Supplementary material for: Exploring the impact of primer length on efficient gene detection via high-throughput sequencing
Source: Nat Commun. 2024 Jul 12;15:5858. doi: 10.1038/s41467-024-49685-0 (PMC11245535; doi:10.1038/s41467-024-49685-0)
Supplement: Supplementary file 5 — Reporting Summary [file 41467_2024_49685_MOESM5_ESM.pdf]

nature portfolio

Corresponding author(s): Damian Wollny
Last updated by author(s): May 5, 2024

Reporting Summary

Nature Portfolio wishes to improve the reproducibility of the work that we publish. This form provides structure for consistency and transparency in reporting. For further information on Nature Portfolio policies, see our Editorial Policies and the Editorial Policy Checklist.

Statistics

For all statistical analyses, confirm that the following items are present in the figure legend, table legend, main text, or Methods section.

n/a	Confirmed	
☐	☒	The exact sample size (*n*) for each experimental group/condition, given as a discrete number and unit of measurement
☐	☒	A statement on whether measurements were taken from distinct samples or whether the same sample was measured repeatedly
☐	☒	The statistical test(s) used AND whether they are one- or two-sided<br>*Only common tests should be described solely by name; describe more complex techniques in the Methods section.*
☒	☐	A description of all covariates tested
☐	☒	A description of any assumptions or corrections, such as tests of normality and adjustment for multiple comparisons
☐	☒	A full description of the statistical parameters including central tendency (e.g. means) or other basic estimates (e.g. regression coefficient) AND variation (e.g. standard deviation) or associated estimates of uncertainty (e.g. confidence intervals)
☐	☒	For null hypothesis testing, the test statistic (e.g. *F*, *t*, *r*) with confidence intervals, effect sizes, degrees of freedom and *P* value noted<br>*Give P values as exact values whenever suitable.*
☒	☐	For Bayesian analysis, information on the choice of priors and Markov chain Monte Carlo settings
☒	☐	For hierarchical and complex designs, identification of the appropriate level for tests and full reporting of outcomes
☒	☐	Estimates of effect sizes (e.g. Cohen's *d*, Pearson's *r*), indicating how they were calculated

Our web collection on statistics for biologists contains articles on many of the points above.

Software and code

Policy information about availability of computer code

Data collection	Data was generated by Illumina sequencing.
Data analysis	seqtk (version 1.0-r82-dirty)<br>BBDuk program of the BBMap suite of tools (version 38.93)<br>STAR (version 2.7.8a)<br>RSEM tool (version 1.3.1)<br>R (version 4.2.2)<br>R packages: ggplot2 (version 3.4.2), ensembldb (version 2.20.2), UpSetR (version 1.4.0), TissueEnrich (version 1.14.0), ComplexHeatmap (version 2.15.4), BioanalyzeR (version 0.10.1), stats (version 4.3.3), car (version 3.1-2), rstatix (version 0.7.2), effsize (version 0.8.1)<br>DAVID Functional Annotation tool (DAVID Version 2021, The DAVID Knowledgebase (v2023q3)<br>Custom code available at: jumicheel/random_priming (github.com)

For manuscripts utilizing custom algorithms or software that are central to the research but not yet described in published literature, software must be made available to editors and reviewers. We strongly encourage code deposition in a community repository (e.g. GitHub). See the Nature Portfolio guidelines for submitting code & software for further information.

Data

Policy information about availability of data

All manuscripts must include a data availability statement. This statement should provide the following information, where applicable:

- Accession codes, unique identifiers, or web links for publicly available datasets
- A description of any restrictions on data availability
- For clinical datasets or third party data, please ensure that the statement adheres to our policy

The raw sequencing reads generated in this study have been deposited in the NCBI BioProject database (https://www.ncbi.nlm.nih.gov/bioproject) under accession code PRJNA983129 (Human brain RNA-seq data) [https://www.ncbi.nlm.nih.gov/bioproject/PRJNA983129] and PRJNA1041084 (Vero cell RNA-seq data) [https://www.ncbi.nlm.nih.gov/bioproject/PRJNA1041084]. The processed sequencing data and electrophoresis data are available at jumicheel/random_priming (github.com). The data shown in this study are provided in the Source Data file. The human genome sequence used in this study is available at http://ftp.ebi.ac.uk/pub/databases/gencode/Gencode_human/release_38/GRCh38.primary_assembly.genome.fa.gz. The human genome annotation used in this study is available at http://ftp.ebi.ac.uk/pub/databases/gencode/Gencode_human/release_38/gencode.v38.primary_assembly.annotation.gtf.gz. ENSEMBL version 105 human gene and transcript annotation (annotation hub 'AH98047') is additionally provided on jumicheel/random_priming (github.com). The AGM genome sequence used in this study is available at http://ftp.ensembl.org/pub/release-105/fasta/chlorocebus_sabaeus/dna/Chlorocebus_sabaeus.ChlSab1.1.dna.toplevel.fa.gz. The AGM genome annotation used in this study is available at http://ftp.ensembl.org/pub/release-105/gtf/chlorocebus_sabaeus/Chlorocebus_sabaeus.ChlSab1.1.105.gtf.gz. The SARS-COV2 genome sequence used in this study is available at http://ftp.ensemblgenomes.org/pub/viruses/fasta/sars_cov_2/dna/Sars_cov_2.ASM985889v3.dna.toplevel.fa.gz. The SARS-COV2 genome annotation used in this study is available at http://ftp.ensemblgenomes.org/pub/viruses/gtf/sars_cov_2/Sars_cov_2.ASM985889v3.101.gtf.gz. The data frame `brain_all_genes` available at jumicheel/random_priming (github.com) and contains all protein-coding genes detected in the brain from the Human Protein Atlas (downloaded on 11 May 2023).

Research involving human participants, their data, or biological material

Policy information about studies with human participants or human data. See also policy information about sex, gender (identity/presentation), and sexual orientation and race, ethnicity and racism.

Reporting on sex and gender	Research in this study does no involve human participants, their data or biological material.<br>Both terms sex and gender are not mentioned within this study.
Reporting on race, ethnicity, or other socially relevant groupings	No socially constructed or socially relevant categorization variables are mentioned within this study.
Population characteristics	There are no human research participants in this study.
Recruitment	No participants were recruited for this study.
Ethics oversight	Research in this study does no involve human participants, their data or biological material.

Note that full information on the approval of the study protocol must also be provided in the manuscript.

Field-specific reporting

Please select the one below that is the best fit for your research. If you are not sure, read the appropriate sections before making your selection.

☒ Life sciences ☐ Behavioural & social sciences ☐ Ecological, evolutionary & environmental sciences

For a reference copy of the document with all sections, see nature.com/documents/nr-reporting-summary-flat.pdf

Life sciences study design

All studies must disclose on these points even when the disclosure is negative.

Sample size	No statistical method was used to pre-determine sample sizes.<br>In this technical study, we used three technical replicates per condition per individual experiment. Three independent sequencing experiments were performed with varying input materials or varying sequencing depth to confirm the effects that we found under variable conditions.<br>The number of three technical replicates was chosen as it is common in this field of research and as it represents the minimal number of

	replicates to detect possible experimental outliers. Additionally we had to consider to sequence all samples per independent experiment together to avoid batch effects while maintaining a reasonable sequencing depth.<br>In addition, the observed effect was confirmed in a fourth independent experiment with a different method (fragment size analysis) and an additional input material with again three technical replicates per condition.
Data exclusions	No data was excluded from the analyses.
Replication	The technical study was replicated in three independent experiments with variations in sequencing depth and input material in order to generalize the observed effects.
Randomization	Randomization was not relevant to our study because investigators were comparing sequencing library generation under well controlled conditions<br>(variable random primer lengths). No human or animal subjects were used in the study. Randomization is not generally used in this field.
Blinding	Blinding was not relevant to our study because the results are quantitative and did not require subjective judgment or interpretation. Blinding is not typically used in the field.

Reporting for specific materials, systems and methods

We require information from authors about some types of materials, experimental systems and methods used in many studies. Here, indicate whether each material, system or method listed is relevant to your study. If you are not sure if a list item applies to your research, read the appropriate section before selecting a response.

Materials & experimental systems

n/a	Involved in the study
☒	☐ Antibodies
☐	☒ Eukaryotic cell lines
☒	☐ Palaeontology and archaeology
☒	☐ Animals and other organisms
☒	☐ Clinical data
☒	☐ Dual use research of concern
☒	☐ Plants

Methods

n/a	Involved in the study
☒	☐ ChIP-seq
☒	☐ Flow cytometry
☒	☐ MRI-based neuroimaging

Eukaryotic cell lines

Policy information about cell lines and Sex and Gender in Research

Cell line source(s)	VERO cells (Cytion, cat. no. 605372) were cultured and processed by cooperation partners Ruben Rose (Institute of Infection Medicine, Kiel University and University Hospital Schleswig-Holstein, Kiel, Germany) and Andi Krumbholz (Institute of Infection Medicine, Kiel University and University Hospital Schleswig-Holstein, Kiel, Germany; Labor Dr Krause und Kollegen MVZ GmbH, Kiel, Germany) as indicated in the Acknowledgements section. Their experimental part is provided as a reference in the Methods section. The authors of this study were provided with the readily isolated RNA of the VERO cells.
Authentication	The cell line was not authenticated.
Mycoplasma contamination	The cell line was not tested for mycoplasma contamination.
Commonly misidentified lines<br>(See ICLAC register)	The VERO cell line used in this study is not reported to be commonly misidentified in the ICLAC register.

Plants

Seed stocks	*Report on the source of all seed stocks or other plant material used. If applicable, state the seed stock centre and catalogue number. If plant specimens were collected from the field, describe the collection location, date and sampling procedures.*
Novel plant genotypes	*Describe the methods by which all novel plant genotypes were produced. This includes those generated by transgenic approaches, gene editing, chemical/radiation-based mutagenesis and hybridization. For transgenic lines, describe the transformation method, the number of independent lines analyzed and the generation upon which experiments were performed. For gene-edited lines, describe the editor used, the endogenous sequence targeted for editing, the targeting guide RNA sequence (if applicable) and how the editor was applied.*
Authentication	*Describe any authentication procedures for each seed stock used or novel genotype generated. Describe any experiments used to assess the effect of a mutation and, where applicable, how potential secondary effects (e.g. second site T-DNA insertions, mosiacism, off-target gene editing) were examined.*
